# Clinical Context Generation for Imaging: A Design Thinking-based Analysis of a Pilot Project

**DOI:** 10.7759/cureus.3063

**Published:** 2018-07-30

**Authors:** Faiq Shaikh, Anna Von Reden, Brian Kolowitz, Omer Awan, Rasu Shrestha

**Affiliations:** 1 Institute of Computational Health Sciences, University of California San Francisco, San Francisco, USA; 2 UPMC Enterprises, University of Pittsburgh School of Medicine, Pittsburgh, USA; 3 Department of Radiology, Dartmouth Hitchcock Medical Center, New Hampshire, USA

**Keywords:** imaging, design thinking, clinical context generation

## Abstract

Design Thinking is a method for the practical, creative resolution of problems using the strategies used during the process of designing. It is increasingly being used in Medical enterprise to develop a solution-based approach to identify ambiguous problems and create alternative paths to the solution. We faced several challenges in the development of a clinical context generation tool and in this article, we retrospectively assess the usefulness of a Design Thinking approach had it been applied to a project related to Medical Imaging-related clinical context generation.

## Introduction

As healthcare in the United States undergoes a shift from a volume- to value-based system and from an individual to a population-based model, there are several aspects of this transition that require a dedicated study. Adding value by improving quality and reducing cost can be accomplished using a spectrum of changes ranging from minor tweaks to major overhauls [1]. The objective is to redesign and not just restructure or re-engineer the product. This requires an empathic approach to the situation at hand, which includes understanding health statistics, economic constraints, market needs, and government policy. Design Thinking enables us to have an understanding of how various healthcare models such as urgent care clinics, hospitals, laboratory testing sites, or rehabilitation and skilled nursing facilities can adapt to the challenges in the environment [2]. Design Thinking also creates opportunities for organizations to thrive and provide solutions to changing requirements, for instance, value-based-payment, regulatory changes, and patient-centered care. These solutions are vast and can include improvement opportunities in mobile health solutions, interactive patient portals, and population health-centric wellness programs [3,4].

## Technical report

Our project was aimed at providing radiologists unified access to clinical content pertinent to their patients’ imaging exams, regardless of information source system. The application quickly became a cautionary tale, illustrating the consequences of beginning product development before Design Thinking had sufficient time to complete the “what is” step of problem framing. From start to finish, the application suffered from identity issues that stemmed from attempts to balance strong user experience against non-trivial clinical risk, pressure to deploy, and the value of the desired research data. Ultimately, the pilot did not proceed forward, but the lessons learned served to inform several subsequent design and product efforts in the same domain, to greater success. This case study is a retrospective analysis of designed artifacts and learned experiences, leveraging the Gibbs Reflective Model as an approach to structure the analysis.

Hypothesis

This pilot began as a means of addressing this identified need in the workflow, with the subgoal of collecting additional electronic medical record (EMR) data usage statistics to drive the development of more intelligent systems. However, as we will discuss, the requirements dictated by these goals were not always in alignment, and, neither goal had been appropriately defined when development started. Though we feel the hypothesis is still valid and prevalent in diagnostic imaging, better integration of Design Thinking upfront could have situated the team and the pilot to meet their goals.

Overview

A high number of these studies arrive on radiologists’ workstations with little-to-no additional clinical context about the patient. At times the patient lacks extensive medical history, but in many other cases, the lack of context can be attributed a) to access barriers between radiologists and the electronic record, and/or b) lack of emphasis upon the clinical details within the broader record that prompted the imaging event. The transition to a value-based care paradigm opens many questions about appropriate or even mandated use of the clinical record in imaging interpretation. Imaging 3.0 describes a blueprint for better care, but the stepping-stones to higher quality are not yet fully defined [5].

What happened?

**Figure 1:**
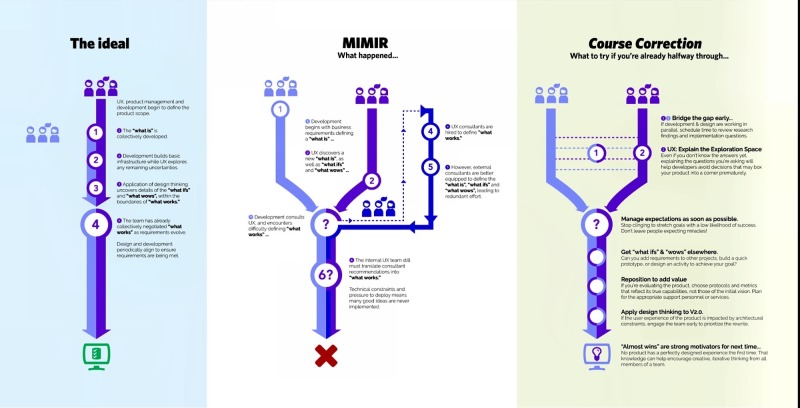
Pilot project and Design Thinking-based course correction. The pilot project outlines a scenario where a lack of design thinking at the outset had significant downstream impacts on the success of the project. To the right we share several takeaways on how to recognize similar situations early, and how to better integrate design thinking into in-progress development efforts.

From the moment the green light was given to begin work on our proof of concept (PoC), there was a conflict between the two previously mentioned goals for its use. As is often the case in a commercial software development environment—especially those subscribing to Agile methodologies—user experience (UX) design and development began their work simultaneously, with related but non-unified missions. As the need for broader Design Thinking was gradually realized, it was integrated into the development process. This created new challenges for the team to overcome in its effort to deploy the application on time (see Figure 1).

Phase 1: Decoupled from Design Thinking

1. Initial Development

Data aggregation and display was by no means a new concept at the start of this project. At that point in time, our organization already had several examples of software that consolidated certain pieces of traditionally distributed data, including a proprietary plugin that shows radiologists extra exam and patient details, and Intelligent Document Repository capable of aggregating and tagging content from multiple EMR software. However, both of these systems had several limitations impacting workflow efficiency. The existing proprietary software relied on laborious and sometimes redundant data entry by technologists. The clinical data repository system in place, despite having a strong back-end, had no clinical user interface (UI), much less one tailored to a radiologist workflow. The first wave of the development effort attempted to prove quickly that technical hurdles to EMR content consolidation could be overcome, and to develop a useable interface to support radiology-specific workflows, first as a standalone application, but eventually as part of a larger commercial product. This remained Goal 1 for the course of the project.

2. Initial UX Research

The goals of the development team grew from requirements provided by the business with minimal input from the UX team, who, at the time were occupied with defining the broader radiology workflows of the product suite. With this product definition came valuable insights about the nature of the data desired by radiologists, and the situationally dependent value of data visualization. For example, initial observations by business analysts revealed that despite non-trivial technical obstacles, radiologists at UPMC were looking to the EMR for additional patient information quite often. This was primarily for documents describing recent patient encounters with the referring physician (Office Notes, Progress Notes, Consults, H&Ps, ED Notes), or for records of surgery and results of subsequent pathology. However, radiologists did not always need all of this content every time they read an exam. The UX team subsequently conducted discovery sessions using a number of common design research methodologies—card sorting, demography surveys, contextual inquiry—to better understand how pertinent data changed with the stage of the workflow and with the patient’s individual clinical situation.

Phase 2: Incorporating Design Thinking

As we tried to catch up on lost time in terms of design thinking application, we realized that user evaluations led to a large backlog of UI adjustments and that reorganizing established EMR categories into a new model was actually time lost, if the radiologist still ultimately had to search for content. From this research, the UX team along with the business began to flesh out a new set of goals and requirements that fall neatly within the Design Thinking structure.

What Is?

A system that goes one step beyond simple EMR consolidation and attempts to show only the subset of data relevant to particular workflow and clinical scenarios (e.g., interpretation =/= protocoling, inpatient =/= outpatient, and cancer =/= trauma).

What If?

What if we could gather a large enough dataset that we could determine the criteria for data relevancy down to the level of individual patients, and the contents of individual documents? What if we could use information about radiologists’ prior usage to inform or predict future radiologists’ needs in similar situations? What works? We needed a system that intuitively presented a synopsis of the most high-yield information relevant to the case being studied, in a seamless fashion and without burdening the system resources or the user (radiologist).

What Wows?

How does one use interaction design to encourage volume-pressured radiologists to use a clinical synopsis that provides key information on the case? How do you encourage behavior change in the radiology workflow so that feedback to support machine learning becomes accepted or even expected? These ideas culminated in a new, secondary Goal 2 for the pilot: to incorporate a means of capturing data usage and the value of data to the radiologist within the context of an exam. UX consultant team hired to investigate changes to existing application that would support Goals 1 and 2 (How can a pilot be a production application and a research tool simultaneously?).

Phase 3: Designing for production acceptance (What works?)

UX consultant team proposed an ideal system, internal team had to deconstruct it to arrive at the minimal viable product for radiologist acceptance. Unfortunately, development had already built most of the back-end and UI infrastructure, so adjusting features was challenging if not impossible. We couldn’t change the structure of the navigation or the way it was accessed (legibility issues), and there were issues accurately capturing use data through implicit measures like timestamps. There was pressure for data to be clinically valid to enable use for Goal 1 in a production environment, despite the shift in focus leaning more and more toward Goal 2. Deployment deadlines, business promises to deliver an application, lead to compromises in user experience. Ultimately, the structure of the captured data in the backend was not designed to allow for scalable analysis, nor tailored to answer specific research questions. Application needed significant user hand-holding, research protocols, environment maintenance, and data analysis that were not properly resourced because the pilot was framed as a production app.

## Discussion

In the realm of Medical Imaging, as we learned, there are many opportunities to redesign the current models that are outdated and poorly equipped to tackle the challenges of a value-based model. This provides a tremendous opportunity for us to incorporate principles of Design Thinking to facilitate the transition of volume- to value-based healthcare.

There is a fine line between valuable and perfect data which is dependent on the conclusion you’re trying to draw. We had activities targeted at both exploratory (Goal 1) and confirmatory (Goal 2) research. Belated use of design consultant resources as an attempt at solving “What works”, when consultants are typically more equipped to answer “What is”, “What if”, “What wows”, and most issues, could have been avoided by properly defining these at the start of the project. Value may have been easier to measure and prove if the data model had been better defined; if we had put more time into thinking through the research questions and understanding how they interrelated not only in terms of data value but timing we would have done things differently. In keeping with the lessons learned, we will choose more appropriate activities (e.g., paper prototype, deeper competitive analysis, scenario definition) over building the PoC early on. We have already developed a stronger distinction between R&D initiatives and productization initiatives in project definition and resource assignment, and improved the lead time for transforming PoCs into commercially viable and clinically robust software. We will adopt a fail-fast, fail-often approach to R&D initiatives: carve out support structures that allow design and development to test concepts but permissibly abandon those that do not work. New IRCC PoCs have been built to answer smaller, bite-sized questions (e.g., what caused the physician to order this exam? instead of what data is helpful to radiologists?), and the form of the UI follows their research function.

It is critically important to understand the role of Design Thinking and the value it brings to product design and commercialization. It is necessary to identify projects that could benefit from its application. The difficult question here is how do we gracefully accept when we have made the wrong choice, but continue to innovate, particularly in a healthcare environment that is rightfully concerned with patient safety but consequently hyper-averse to risk?

Designers have been traditionally recruited with a focus in usability and/or visual design near the end of the production cycle in order to make the engineered product more palatable to the end-user, which may be translated into a better-looking patient portal or website for a healthcare facility [6]. However, it is important to understand that the tools of design need to be considered at the very start of contemplating an idea and need to be incorporated throughout a project to ensure the product or service resonates with the market needs. In contrast to graphic designers who are employed at the end of a project, human-centered designers use a creative approach to solve problems at all stages in the product life-cycle [7,8]. In addition, the need to improve the overall user experience and more importantly the intended outcome of the project should also be acknowledged.

It is important to address the prevalent belief system of the group of people who are the targeted user of the designed product. This provides tremendous insight into the way the product or service features affect human behavior and is interwoven with the challenges that are faced. Furthermore, it must be recognized that even though the initial steps to foster novel ideas of design framework encourage open-minded and unconventional thinking, the final application needs to be approached pragmatically in order to ensure its translation into a viably executed product [8]. There has been a recent focus on utilizing Design Thinking as a tool to help us transition into this new paradigm in healthcare [9]. This helps the thinkers in the field to create novel ways of approaching complex tasks and catalyze change. It is still a novel concept in healthcare; however, the awareness of the role of Design Thinking in facilitating and catalyzing the innovative transformation in medicine is increasing. This trend of using Design Thinking approaches to increase quality and efficiency of healthcare delivery is seen in companies like IDEO (Palo Alto, CA, USA) that worked with Kaiser Permanente (Oakland, CA, USA) and found ways to bring the time it takes nurses to change shift by going through a design-based exploration. Another innovative company in this space is Jump Associates (San Mateo, CA, USA) that is working with Stanford University on various design thinking projects.

## Conclusions

The importance of Design Thinking in inducing change in the status quo in radiology practice is becoming evident and will soon be inescapable. Design Thinking makes the otherwise arduous task of this paradigm shift to value-based, patient-centric and outcomes-focused healthcare model more intuitive, palatable and inclusive. It needs a forward-thinking, agile approach that is willing to recognize the challenge at hand, approach it creatively, put its assumptions to test and catalyze change.
